# Decreasing and stabilising trends of antimicrobial consumption and resistance in *Escherichia coli* and *Klebsiella pneumoniae* in segmented regression analysis, European Union/European Economic Area, 2001 to 2018

**DOI:** 10.2807/1560-7917.ES.2019.24.46.1900656

**Published:** 2019-11-14

**Authors:** Germán Peñalva, Liselotte Diaz Högberg, Klaus Weist, Vera Vlahović-Palčevski, Ole Heuer, Dominique L Monnet, ESAC-Net study group, EARS-Net study group

**Affiliations:** 1 Department of Infectious Diseases, Microbiology and Preventive Medicine, Institute of Biomedicine of Seville (IBiS), University Hospital Virgen del Rocio, CSIC, University of Seville, Spain; 2 European Centre for Disease Prevention and Control, Solna, Sweden; 3 Department of Clinical Pharmacology, University Hospital Rijeka / Medical Faculty and Faculty of Health Studies, University of Rijeka, Rijeka, Croatia; 4 ESAC-Net study group participants are listed at the end of the article; 5 EARS-Net study group participants are listed at the end of the article

**Keywords:** Antimicrobial consumption, antimicrobial resistance, *Escherichia coli*, *Klebsiella pneumoniae*, bacterial infections, defined daily dose, fluoroquinolone, third-generation cephalosporin, carbapenem

## Abstract

Investments to reduce the spread of antimicrobial resistance (AMR) in the European Union have been made, including efforts to strengthen prudent antimicrobial use. Using segmented regression, we report decreasing and stabilising trends in data reported to the European Surveillance of Antimicrobial Consumption Network and stabilising trends in data reported to the European Antimicrobial Resistance Surveillance Network. Our results could be an early indication of the effect of prioritising AMR on the public health agenda.

Antimicrobial resistance (AMR) is one of the main public health challenges worldwide. In the European Union (EU) and European Economic Area (EEA), 33,000 deaths are estimated to be attributed to infections with AMR bacteria annually [1]. Ensuring prudent antimicrobial use is key to an effective response to AMR, as antimicrobial use exerts ecological pressure on bacteria and contributes to the emergence and selection of resistant bacteria. Here, we describe trends in antimicrobial consumption of fluoroquinolones, third-generation cephalosporins and carbapenems between 2001 and 2018 and for AMR in *Escherichia coli* and *Klebsiella pneumoniae*.

## Initiatives targeting antimicrobial resistance in EU/EEA

The European Antibiotic Awareness Day, a European health initiative and annual event coordinated by the European Centre for Disease Prevention and Control (ECDC), was initiated in 2008 to support EU/EEA countries in their efforts to prevent and control AMR by raising awareness about prudent use of antibiotics [2]. Several EU and global initiatives followed, all prompting action at EU/EEA and at country level. Among these were: (i) a first EU Action Plan against the rising threat from AMR in 2011 [3], (ii) a World Health Organization Global Action Plan on AMR in 2015 [4], (iii) EU Guidelines for the prudent use of antimicrobials in human health [5] (iv) a new European One Health Action Plan against AMR in 2017 [6], and (v) various Council Conclusions adopted by EU Member States between 2008 and 2019 [7]. If successful, these efforts should be reflected in the trends of antimicrobial consumption and of corresponding AMR levels in the EU/EEA.

## Analysis of antimicrobial consumption and antimicrobial resistance trends

To examine trends, we analysed population-weighted EU/EEA-wide data from the European Surveillance of Antimicrobial Consumption Network (ESAC-Net, formerly ESAC) and the European Antimicrobial Resistance Surveillance Network (EARS-Net, formerly EARSS). Using joinpoint version 4.7.0.0 (National Cancer Institute, Bethesda, United States), we performed a regression analysis of data reported to ESAC-Net/ESAC on defined daily doses (DDDs) as listed in the Anatomical Therapeutic Chemical (ATC) Index for 2019 [8] on the consumption of fluoroquinolones (ATC group J01MA), third-generation cephalosporins (ATC group J01DD) and carbapenems (ATC group J01DH) in the community and hospital sector between 2001 and 2018. Using data from EARS-Net/EARSS, corresponding AMR percentages in invasive *E. coli* (2002–18) and *K. pneumoniae* (2006–18) isolates were analysed with the same joinpoint methodology. *E. coli* and *K. pneumoniae* are among the pathogens contributing most to the burden of AMR in the EU/EAA [1] and for which AMR levels have increased substantially during the last decades [9-12]. Joinpoint regression is a time series analysis technique that is especially useful when the slope of the regression function is expected to change over time, as it allows the analyses to identify the points in time at which trends change and to characterise segments in the time series [13]. Materials and methods are detailed in the Supplementary Material, including an additional sensitivity analysis.

## Trends in EU/EEA population-weighted antimicrobial consumption

**Figure 1:**
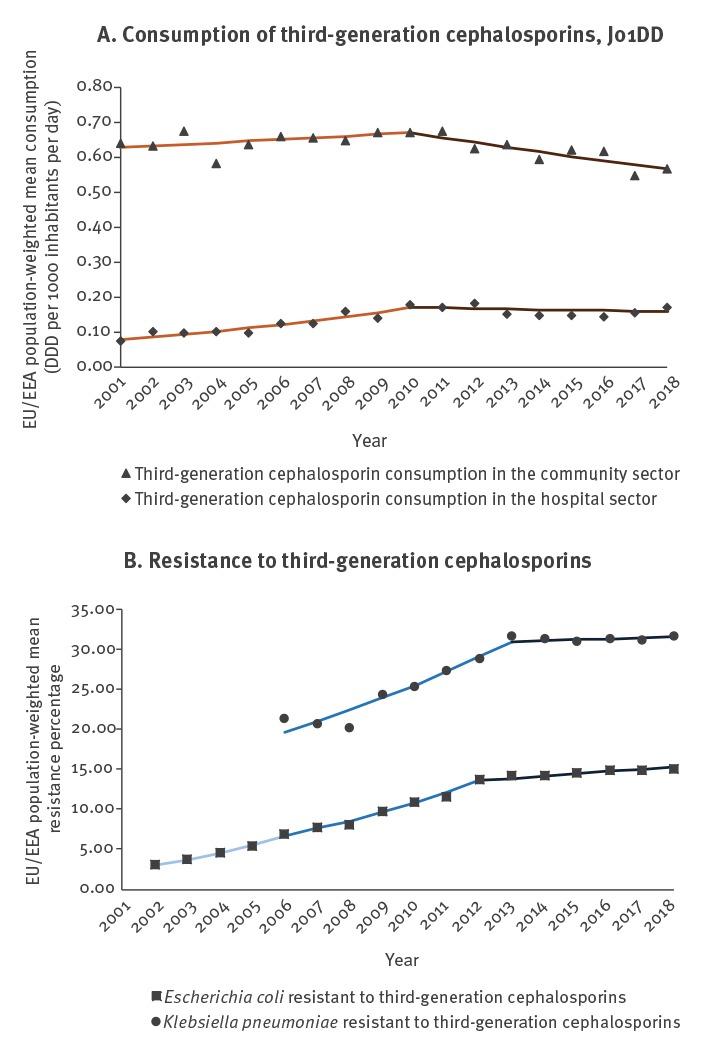
Consumption of third-generation cephalosporins by sector and third-generation cephalosporin resistance by microorganism, including detected trend segments, EU/EEA population-weighted means, 2001–2018

**Figure 2:**
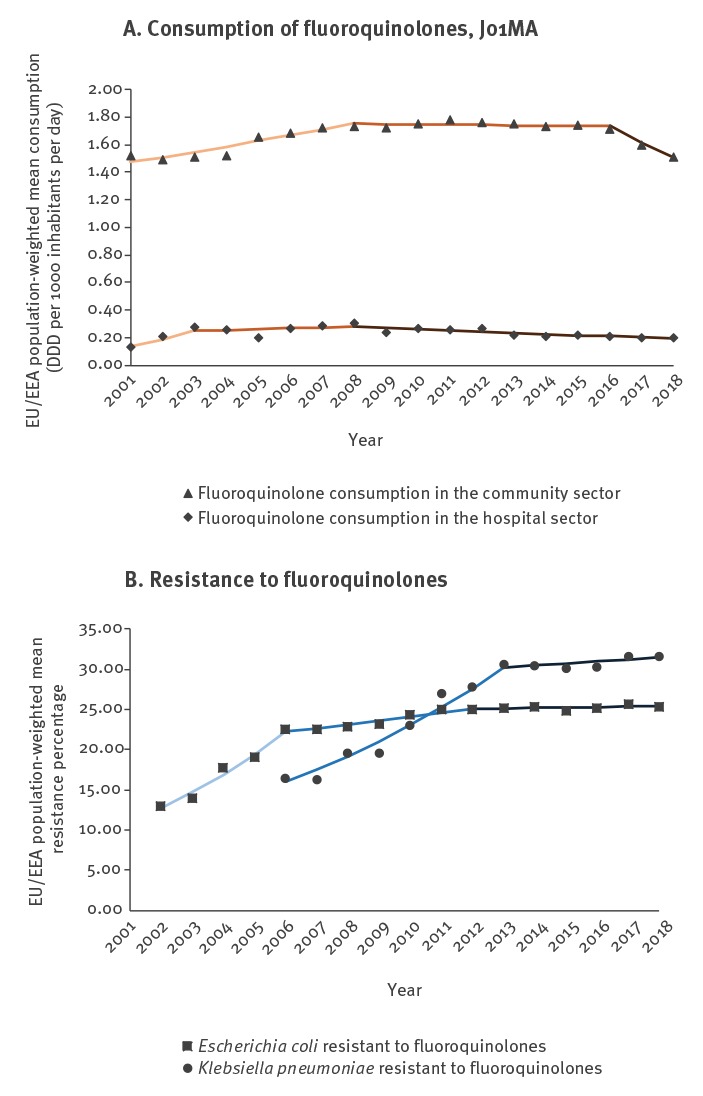
Consumption of fluoroquinolones by sector, and fluoroquinolone resistance by microorganism, including detected trend segments, EU/EEA population-weighted means, 2001–2018

**Figure 3:**
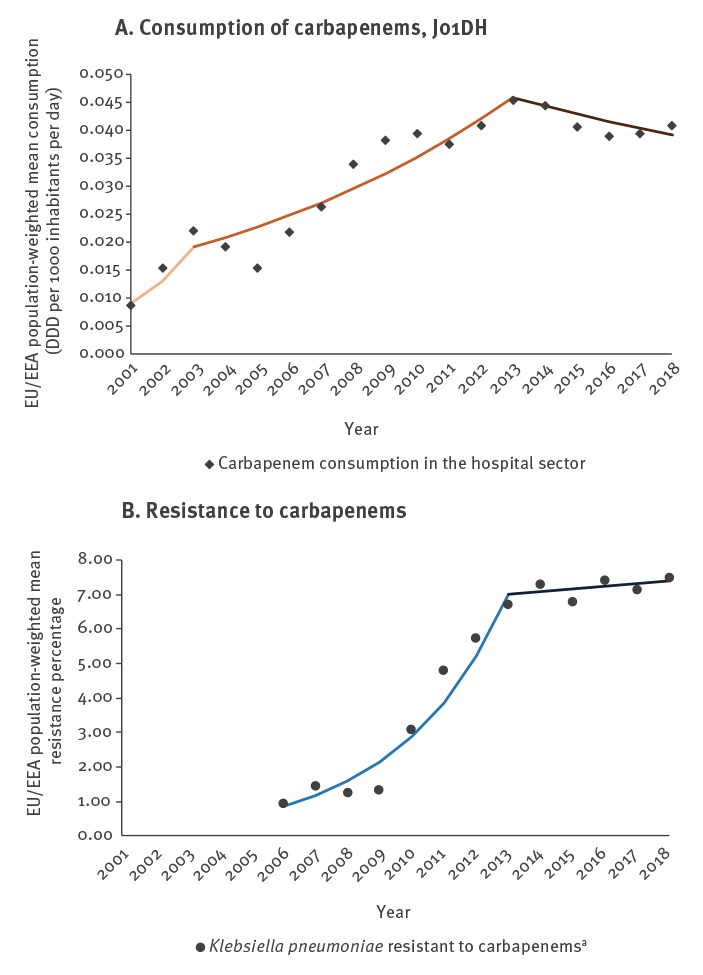
Consumption of carbapenems in the hospital sector and carbapenems resistance in *Klebsiella pneumoniae*, including detected trend segments, EU/EEA population-weighted means, 2001–2018

For antimicrobial consumption, the joinpoint model identified two separate trend segments for third-generation cephalosporin consumption (Table 1, Figure 1 and three separate trend segments for fluoroquinolone and carbapenem consumption during 2001–2018 (Table 1, Figure 2 and Figure 3). For the purpose of this article, we use the terms ‘increase’ or ‘decrease’ when the trend of the segment was statistically significant (p ≤ 0.05) and we used the term ‘stable’ when there was no statistically significant trend (p > 0.05).

**Table 1:** Joinpoint regression analysis of trends: EU/EEA population-weighted mean consumption expressed in DDD per 1000 inhabitants per day, per antimicrobial group and sector, ESAC-Net/ESAC, 2001–2018

Segment	Lower endpoint		Upper endpoint		APC		
	Year	95% CI	Year	95% CI	APC (%)	95% CI	P value
**Third-generation cephalosporin consumption (ATC group J01DD)**							
**Community sector**							
All years	2001	NA	2018	NA	**-0.6**	**-1.2 to -0.1**	**0.033**
Segment 1	2001	NA	2010	2008 to 2013	+ 0.7	-0.04 to 1.5	0.062
Segment 2	2010	2008 to 2013	2018	NA	**-2.1**	**-3.0 to -1.2**	**< 0.001**
**Hospital sector**							
All years	2001	NA	2018	NA	**+ 4.3**	**0.8 to 7.8**	**0.015**
Segment 1	2001	NA	2010	2003 to 2015	**+ 9.0**	**3.8 to 14.5**	**0.002**
Segment 2	2010	2003 to 2015	2018	NA	-0.9	-6.3 to 4.9	0.746
**Fluoroquinolone consumption (ATC group J01MA)**							
**Community sector**							
All years	2001	NA	2018	NA	+ 0.1	-0.8 to 1.0	0.780
Segment 1	2001	NA	2008	2005 to 2013	**+ 2.5**	**1.5 to 3.6**	**< 0.001**
Segment 2	2008	2005 to 2013	2016	2012 to 2016	-0.1	-1.2 to 0.9	0.760
Segment 3	2016	2012 to 2016	2018	NA	**-6.8**	**-12.8 to -0.4**	**0.040**
**Hospital sector**							
All years	2001	NA	2018	NA	+ 2.0	-1.9 to 5.9	0.318
Segment 1	2001	NA	2003	2003 to 2005	**+ 34.3**	**1.0 to 78.5**	**0.044**
Segment 2	2003	2003 to 2005	2008	2006 to 2016	+ 2.1	-6.2 to 11.2	0.598
Segment 3	2008	2006 to 2016	2018	NA	**-3.6**	**-5.5 to -1.6**	**0.003**
**Carbapenem consumption (ATC group J01DH)**							
**Hospital sector**							
All years	2001	NA	2018	NA	**+ 9.2**	**3.8 to 14.7**	**0.001**
Segment 1	2001	NA	2003	2003 to 2012	**+ 46.9**	**4.2 to 107.0**	**0.032**
Segment 2	2003	2003 to 2012	2013	2006 to 2016	**+ 9.2**	**4.4 to 14.2**	**0.001**
Segment 3	2013	2006 to 2016	2018	NA	-3.1	-12.4 to 7.2	0.500

APC: annual percentage change; CI: confidence interval; DDD: defined daily doses; ESAC-Net/ESAC: European Surveillance of Antimicrobial Consumption Network/European Surveillance Antimicrobial Consumption project; NA: not applicable.

Statistically significant trends are shown in bold.

DDD: defined daily doses; EU/EEA: European Union and European Economic Area.

Antimicrobial consumption trend segments are shown in red. AMR trend segments are shown in blue**.**

DDD: defined daily doses; EU/EEA: European Union and European Economic Area.

Antimicrobial consumption trend segments are shown in red. AMR trend segments are shown in blue**.**

DDD: defined daily doses; EU/EEA: European Union and European Economic Area.

^a^ Carbapenem resistance in *E. coli* was not included. Because carbapenem resistance is rare in *E. coli* isolates in the EU/EEA, carbapenem susceptibility is in many cases not routinely determined for this species in clinical microbiology laboratories.

Antimicrobial consumption trend segments are shown in red. AMR trend segments are shown in blue**.**

The most recent trend segments, starting between 2008 and 2016 depending on the antimicrobial group and sector, all showed stable or decreasing trends, while segments covering earlier years showed larger variation (Table 1, Figures 1-3). For consumption of third-generation cephalosporins in hospital sector (Table 1, Figure 1) and carbapenems in the hospital sector (Table 1, Figure 3), the most recent segments showed stable levels whereas previous segments showed increasing trends. For consumption of third-generation cephalosporins in the community (Table 1, Figure 1) as well as consumption of fluoroquinolones in both sectors (Table 1, Figure 2), the most recent segments showed a decreasing trend.

## Trends EU/EEA-population weighted antimicrobial resistance percentages

The joinpoint model identified three separate trend segments for *E. coli* (Table 2, Figures 1-2), and two trend segments for *K. pneumoniae* (Table 2, Figures 1-3). The most recent segments, starting between 2012 and 2013 depending on the microorganism-antimicrobial group combination, showed more favourable trends, i.e. a stable level (fluoroquinolone resistance in *E. coli* and *K. pneumoniae*, third-generation cephalosporin resistance and carbapenem resistance in *K. pneumoniae*) or an increase but with a smaller slope compared with previous segments (third-generation cephalosporin resistance in *E. coli*) (Table 2). For almost all microorganism/antimicrobial group combinations, the reduction and/or stabilisation of antimicrobial consumption started before or the same year as the trend in AMR stabilised (Figures 1-3).

**Table 2:** Joinpoint regression analysis of trends: EU/EEA population-weighted mean percentage of resistance, by microorganism and antimicrobial group, EARS-Net/EARSS, 2002–2018

Segment	Lower endpoint		Upper endpoint		APC		
	Year	95% CI	Year	95% CI	APC (%)	95% CI	P value
***Escherichia coli***							
**Third-generation cephalosporin resistance**							
All years	2002	NA	2018	NA	**+ 10.6**	**9.7 to 11.5**	**< 0.001**
Segment 1	2002	NA	2006	2005 to 2008	**+ 21.8**	**19.0 to 24.7**	**< 0.001**
Segment 2	2006	2005 to 2008	2012	2011 to 2014	**+ 12.4**	**10.7 to 14.2**	**< 0.001**
Segment 3	2012	2011 to 2014	2018	NA	**+ 2.0**	**0.8 to 3.2**	**0.004**
**Fluoroquinolone resistance**							
All years	2002	NA	2018	NA	**+ 4.4**	**3.8 to 5.0**	**< 0.001**
Segment 1	2002	NA	2006	2005 to 2007	**+ 14.9**	**13.0 to 16.8**	**< 0.001**
Segment 2	2006	2005 to 2007	2012	2009 to 2015	**+ 2.0**	**1.0 to 3.1**	**0.002**
Segment 3	2012	2009 to 2015	2018	NA	+ 0.2	-0.6 to 1.0	0.538
***Klebsiella pneumoniae***							
**Third-generation cephalosporin resistance**							
All years	2006	NA	2018	NA	**+ 4.0**	**2.4 to 5.6**	**< 0.001**
Segment 1	2006	NA	2013	2011 to 2016	**+ 6.7**	**4.6 to 8.8**	**< 0.001**
Segment 2	2013	2011 to 2016	2018	NA	+ 0.4	-2.9 to 3.8	0.809
**Fluoroquinolone resistance**							
All years	2006	NA	2018	NA	**+ 5.8**	**3.1 to 8.6**	**0.001**
Segment 1	2006	NA	2013	2009 to 2016	**+ 9.6**	**5.7 to 13.6**	**< 0.001**
Segment 2	2013	2009 to 2016	2018	NA	+ 0.8	-4.4 to 6.2	0.748
**Carbapenem resistance**							
All years	2006	NA	2018	NA	**+ 19.6**	**10.5 to 29.4**	**< 0.001**
Segment 1	2006	NA	2013	2010 to 2016	**+ 34.8**	**21.4 to 49.7**	**< 0.001**
Segment 2	2013	2010 to 2016	2018	NA	+ 1.0	-14.7 to 19.7	0.891

APC: annual percentage change; CI: confidence interval; EARS-Net/EARSS: European Antimicrobial Resistance Surveillance Network/European Antimicrobial Resistance Surveillance System; NA: not applicable.

Statistically significant trends are shown in bold.

## Discussion

The decreasing or stabilising EU/EEA trends in antimicrobial consumption of fluoroquinolones, third-generation cephalosporins and carbapenems described in this study during the last 6–10 years could be an early signal of the positive effects of antimicrobial stewardship initiatives in EU/EEA countries. Although the ecological study design and different data sources used cannot demonstrate a causal relationship between antimicrobial consumption and AMR, the similar, but less pronounced, stabilising trends or change in slope of AMR percentages are encouraging. Our analysis shows that the increase in AMR in *E. coli* and *K.* *pneumoniae* was most prominent up to 2012/13, after which there is a tendency towards stabilising levels. These trends may be less discernible in shorter time series analyses and underline the usefulness of data retrieved from longer reporting periods.

Our study has several limitations. First, for EARS-Net/EARSS, we could not control for changes in the use of clinical breakpoints over time [14]. However, changes have generally been towards lower breakpoints, which would result in increasing AMR percentages rather than the stabilising trends we report. Second, although there are no indications of major changes in the frequency of blood culture sampling during the study period [15,16], other changes, e.g. in the population under surveillance cannot be excluded and could have influenced the results. For ESAC-Net/ESAC, most countries provided sales data, but a few were only able to provide reimbursement data which do not include antimicrobials dispensed without a prescription or prescribed antimicrobials for which reimbursement was not claimed [17]. Finally, for antimicrobial prescriptions, no information was available on the pathogen and its AMR profile. This could have allowed for identification of the factors and reasons for the observed trends in antimicrobial consumption.

Several other factors than consumption of specific antimicrobial groups may have affected the corresponding AMR trends. The antimicrobial groups included in this study were selected as they are of relevance to treat severe infections caused by *E. coli* and *K. pneumoniae,* but they only contribute to approximately 14% of the total consumption of antibacterials for systemic use (ATC group J01) (Supplementary Material). Combined resistance to multiple antimicrobial groups increased during our study period, especially for *K. pneumoniae* [12]. Consequently, one antimicrobial agent could also select co-resistant isolates*.* In addition, changes in infection prevention and control (IPC) practices in hospitals and other healthcare settings in EU/EEA countries during the study period could have affected AMR trends, in particular for *K. pneumoniae* for which most infections are healthcare-associated. Longitudinal data on IPC in hospitals in the EU/EEA are not available to analyse such potential impact. Finally, the trends presented here represent the population-weighted mean for the EU/EEA as a whole and individual countries may report different long-term trends and changes, therefore, each EU/EEA country should assess its own situation.

## Conclusion

Responding to the increasing public health threat of AMR is a priority in the EU [3,6,7]. The trends of antimicrobial consumption of fluoroquinolones, third-generation cephalosporins and carbapenems and the AMR phenotypes reported here provide an indication that the recent public health efforts promoting prudent antimicrobial use are showing results. Nevertheless, percentages of AMR reported here were comparatively much higher in 2018 than in 2002/06 and trends appear to stabilise or slow down rather than decrease in recent years. The results presented should encourage further efforts aiming to improve antimicrobial stewardship and IPC, which should contribute to reduce AMR in the EU/EEA.

## Group authors

**Authors contributing to the ESAC-Net study group:** Belgium: Eline VANDAEL, Sciensano, Brussels, Belgium; Bulgaria: Ivan N. IVANOV, National Center of Infectious and Parasitic Diseases, Sofia, Bulgaria; Croatia: Marina PAYERL-PAL, Intersectoral Coordination Mechanism for the Control of Antimicrobial Resistance (ISKRA), Croatia; Denmark: Karoline SKJOLD SELLE PEDERSEN, Statens Serum Institut, Copenhagen, Denmark; Estonia: Elviira LINASK, Bureau of Statistics, Department of Post-authorisation Safety, State Agency of Medicines, Estonia; Germany: Birgitta SCHWEICKERT, Robert Koch Institute, Department for nosocomial infections, surveillance of antimicrobial resistance and antimicrobial consumption, Berlin, Germany; Greece: Flora KONTOPIDOU, Hellenic National Public Health Organization, Greece; Hungary: Mária MATUZ, University of Szeged Department of Clinical Pharmacy, Hungary; Finland: Emmi SARVIKIVI, Finnish Institute for Health and Welfare, Helsinki, Finland; France; Philippe CAVALIÉ, Santé Publique France, the French Public Health Agency, Saint-Maurice, France; Ireland: Ajay OZA, Health Service Executive (HSE) Health Protection Surveillance Centre (HPSC), Dublin, Ireland; Iceland: Guðrún SIGMUNDSDÓTTIR, Centre for Health Security and Communicable Disease Control, Directorate of Health, Reykjavík, Iceland; Italy: Filomena FORTINGUERRA, L'Agenzia Italiana del Farmaco (AIFA), Italy; Lithuania: Rolanda VALINTELIENE; Institute of Hygiene, Lithuania; Luxembourg: Marcel BRUCH, Ministry of Health, Luxembourg; Malta: Peter ZARB, National Antibiotic Committee, Malta; Netherlands: Stephanie NATSCH, Department of Pharmacy, Radboud University Medical Center, Nijmegen, the Netherlands; Norway: Hege Salvesen BLIX, Department of Drug Statistics, Norwegian Institute of Public Health, Oslo, Norway; Poland: Anna OLCZAK-PIEŃKOWSKA, National Medicines Institute, Department of Epidemiology and Clinical Microbiology, Warsaw, Poland; Romania: Ionel IOSIF, National Institute of Public Health, Romania; Spain: Maria Teresa ALONSO HERRERAS, The Spanish Agency for Medicine and Health Products (National Action Plan on Antibiotic Resistance), Spain; Sweden: Vendela WIENER, the Public Health Agency of Sweden, Solna, Sweden; Slovenia: Milan ČIŽMAN, University Medical Centre, Department of Infectious Diseases, Ljubljana, Slovenia; Slovakia: Tomáš TESAŘ, Department of Organisation and Management in Pharmacy, Faculty of Pharmacy, Comenius University in Bratislava, Slovakia; United Kingdom: Berit MULLER-PEBODY, Public Health England, London, United Kingdom.

**Authors contributing to the EARS-Net study group:** Belgium: Lucy CATTEAU, Healthcare-associated infections and antimicrobial resistance, Sciensano, Brussels, Belgium; Bulgaria: Stefana Doneva SABTCHEVA, National Oncology Centre - USHATO, Sofia, Bulgaria; Croatia: Arjana TAMBIC ANDRASEVIC, Department of Clinical Microbiology, University Hospital for Infectious Diseases, Zagreb, Croatia; Cyprus: Panagiota MAIKANTI-CHARALAMPOUS, Microbiology Department- National Reference Laboratory for Antimicrobial Resistance Surveillance, Nicosia General Hospital, Nicosia, Cyprus; Czech Republic: Helena ŽEMLIČKOVÁ, Centre for Epidemiology and Microbiology, Prague, Czech Republic/Department of Laboratory Medicine, Third Faculty of Medicine, Charles University, Prague, Czech Republic; Denmark: Ute SÖNKSEN, Statens Serum Institut, Copenhagen, Denmark; Estonia: Marina IVANOVA, East Tallinn Central Hospital Central Laboratory, Tallinn, Estonia; France: Sylvie MAUGAT, Santé Publique France, the French Public Health Agency, Saint-Maurice, France; Finland: Jari JALAVA, Finnish Institute for Health and Welfare, Helsinki, Finland; Germany: Ines NOLL, Robert Koch Institute, Department for nosocomial infections and surveillance of antimicrobial resistance and antimicrobial consumption, Berlin, Germany; Greece: Michalis POLEMIS, Hellenic National Public Health Organization, Athens, Greece; Hungary: Zsolt VEGH, Department of Bacteriology, Mycology and Parasitology National Public Health Center Albert Florian, Budapest, Hungary; Iceland: Karl G. KRISTINSSON, Department of Clinical Microbiology, Landspitali University Hospital and University of Iceland, Reykjavik, Iceland; Ireland: Karen BURNS, Health Service Executive (HSE) Health Protection Surveillance Centre (HPSC), Dublin, Ireland; Italy: Fortunato D'ANCONA, Istituto Superiore di Sanità, Rome, Italy; Latvia: Kate VULĀNE, Centre for Disease Prevention and Control of Latvia, Infectious Diseases Risk Analysis and Prevention Department, Riga, Latvia; Lithuania: Jolanta MICIULEVICIENE, National Public Health Surveillance Laboratory, Vilnius, Lithuania ; Luxembourg: Monique PERRIN, Laboratoire National de Santé, Dudelange, Luxembourg; Malta: Elizabeth Anne SCICLUNA, Mater Dei Hospital, Malta; the Netherlands: Annelot F. SCHOFFELEN, Centre for Infectious Diseases, Epidemiology and Surveillance, National Institute for Public Health and the Environment (RIVM), Bilthoven, The Netherlands; Norway: Gunnar Skov SIMONSEN, Department of Microbiology and Infection Control, University Hospital of North Norway, Tromsø, Norway; Poland: Dorota ŻABICKA, National Medicines Institute, Warsaw, Poland; Portugal: Manuela CANIÇA, National Institute of Health Doutor Ricardo Jorge, Lisboa, Portugal; Romania: Gabriel Adrian POPESCU, Carol Davila University of Medicine and Pharmacy, Bucharest, Romania; Slovakia: Eva SCHRETEROVA, Louis Pasteur University Hospital Kosice, Kosice, Slovakia; Slovenia: Helena RIBIČ, National Institute of Public Health, Ljubljana, Slovenia; Spain: Belén ARACIL, Antibiotics Laboratory, National Centre of Microbiology, Institute of Health ‘Carlos III’, Majadahonda, Madrid, Spain; Sweden: Hanna BILLSTRÖM, Public Health Agency of Sweden, Solna, Sweden; United Kingdom: Julie WILSON, Health Protection Scotland, Glasgow, United Kingdom.

## Supplementary Data

883000 Supplementary Material
