# Supplementary material for: Decreasing and stabilising trends of antimicrobial consumption and resistance in Escherichia coli and Klebsiella pneumoniae in segmented regression analysis, European Union/European Economic Area, 2001 to 2018
Source: Euro Surveill. 2019 Nov 14;24(46):1900656. doi: 10.2807/1560-7917.ES.2019.24.46.1900656 (PMC6864974; doi:10.2807/1560-7917.ES.2019.24.46.1900656)
Supplement: Supplementary Material [file 1900656_HOGBERG_SupplementaryMaterial.pdf]

SUPLEMENTARY MATERIAL

This supplementary material is hosted by Eurosurveillance as supporting information alongside the article 'Decreasing and stabilising trends of antimicrobial consumption and resistance in *Escherichia coli* and *Klebsiella pneumoniae* in segmented regression analysis, European Union/European Economic Area, 2001 to 2018', on behalf of the authors, who remain responsible for the accuracy and appropriateness of the content. The same standards for ethics, copyright, attributions and permissions as for the article apply. Supplements are not edited by Eurosurveillance and the journal is not responsible for the maintenance of any links or email addresses provided therein.

Materials

Antimicrobial consumption data

The European Surveillance of Antimicrobial Consumption Network (ESAC-Net) is a surveillance network that collects antimicrobial consumption data from European (EU) and European Economic Area (EEA) countries. The network is coordinated by the European Centre for Disease Prevention and Control (ECDC) since 2011. ESAC-Net is the continuation European Surveillance of Antimicrobial Consumption (ESAC) project, which was founded in 2001 and coordinated by the University of Antwerp, Belgium. Antimicrobial consumption data are collected using the Anatomical Therapeutic Chemical (ATC) classification system and defined daily dose (DDD) methodology developed by the WHO Collaborating Centre for Drug Statistics Methodology (Oslo, Norway). For the analysis, DDDs listed in the ATC Index for 2019 were used [1].

ESAC-Net reports consumption separately for the community and the hospital sector, but some countries that are not able to split data by sector reported data for both sectors combined (Table S1). Because the overall consumption in the community has been shown to represents around 90% of the total consumption (when expressed in DDD per 1,000 inhabitants per day and reported for the ATC group J01), ESAC-Net reports total consumption as community consumption for those countries not able to split data.

Data on antimicrobial consumption, for the community and for the hospital sector, expressed as DDD per 1,000 inhabitants per day and reported for 2001–2018 were extracted from The European Surveillance System (TESSy) database at ECDC. The following groups were included in the analysis: fluoroquinolones (ATC group J01MA), third-generation cephalosporins (J01DD) and carbapenems (J01DH). For carbapenems, only data from the hospital sector were included in the analysis as this parenterally administered antimicrobial group is very rarely used in the community in nearly all EU/EEA countries [2-3].

In addition, consumption of all antibacterials for systemic use (ATC group J01) was included in a separate analysis (results presented in this supplement), in order to put our results in the perspective of the entire consumption of antibacterials in the community and hospital sectors.

Antimicrobial consumption data reported by EU/EEA countries to ESAC-Net have high population coverage with a vast majority of countries reporting 100% coverage. Data were obtained mainly from the Ministry of Health or the national medicines agencies or national public health institutes [2].

Antimicrobial resistance data

The European Antimicrobial Resistance Surveillance network (EARS-Net) is a surveillance network that collects routine antimicrobial susceptibility testing (AST) data from clinical microbiology laboratories in EU/EEA countries. The network is coordinated by ECDC since 2010. EARS-Net is the continuation the European Antimicrobial Resistance Surveillance System (EARSS), which was founded in 1998 and coordinated by the Dutch National Institute for Public Health and the Environment (RIVM). The reported AST data are ascertained according to agreed protocols [4-5], and the general quality and comparability of the data are evaluated through an annual external quality assessment exercise offered to the participating laboratories.

Data on *Escherichia coli* and *Klebsiella pneumoniae* isolates from blood and cerebrospinal fluid samples were extracted from The European Surveillance System (TESSy) database at ECDC. Group resistance to third-generation cephalosporins was defined as resistance to at least one of the third-generation cephalosporins under surveillance, i.e. ceftriaxone, ceftazidime or cefotaxime. Group resistance to fluoroquinolones was defined as resistance to at least one of the fluoroquinolones under surveillance, i.e. ciprofloxacin, levofloxacin or ofloxacin. Group resistance to carbapenems was defined as resistance to at least one of the carbapenems under surveillance, i.e. imipenem and meropenem. For carbapenem resistance, only data from *K. pneumoniae* was included in the analysis. Because carbapenem resistance is rare in *E. coli* isolates in the EU/EEA, carbapenem susceptibility is in many cases not routinely determined for this species in clinical microbiology laboratories. For this study, isolates were considered as resistant when tested and interpreted as resistant (R) in agreement with the clinical breakpoint criteria used by the local laboratory.

For each microorganism, data were included from the second year of inclusion in surveillance and up until 2018, thus ensuring a maximum length of time series while avoiding inconsistencies related to comparatively lower reporting frequencies during the year of inclusion in surveillance than in subsequent years. An overview of data availability is provided in Table S1.

Details on data collection and data validity are available in the EARS-Net reporting protocol [4] and EARS-Net annual report [6]. In 2018, data validity, reported as sample representativeness was generally assessed as high in a majority of countries. Data collected during the first part of the time series are based on fewer laboratories and with less information on validity. However, as seen in Figure S2, results based on data from all laboratories (as used in the main analyses) show very little difference to data restricted to countries reporting consistently for all years and laboratories providing data for more than 80%.

TABLE S1. Antimicrobial resistance data (reporting period and number of included isolates by microorganism) and antimicrobial consumption data (reporting period by sector), EU/EEA country, 2001–2018.

Country	Antimicrobial resistance data		Antimicrobial consumption data	
	Escherichia coli	***Klebsiella pneumoniae***	**Community**	**Hospital sector**
	Reporting period (Number of included isolates with information about resistance or susceptibility to fluoroquinolones / third-generation cephalosporins)	Reporting period (Number of included isolates with information about resistance or susceptibility to fluoroquinolones / third-generation cephalosporins / carbapenems)	Reporting period	Reporting period
Austria	2002-2018<br>(55687 / 55792)	2006-2018<br>(10947 / 10964 / 9686)	2001-2018	-
Belgium	2002-2018<br>(41310 / 44265)	2009-2018<br>(5409 / 5385 / 5221)	2001-2018	2001-2018
Bulgaria	2002-2018<br>(3241 / 3268)	2006-2018<br>(1508 / 1510 / 1441)	2001-2005*<br>2006-2018	2006-2018
Croatia	2002-2018<br>(14004 / 14080)	2006-2018<br>(3742 / 3766 / 3753)	2001-2018	2001-2018
Cyprus	2003-2018<br>(1936 / 1941)	2006-2018<br>(838 / 838 / 838)	2006-2017*	-

Czech Republic	2002-2018<br>(44556 / 44589)	2006-2018<br>(17499 / 17503 /13101)	2003-2015	-
Denmark	2003-2018<br>(51400 / 43002)	2006-2018<br>(11658 / 9767 / 8658)	2001-2018	2001-2018
Estonia	2002-2018<br>(5604 / 5533)	2006-2018<br>(1292 / 1319 / 1116)	2001*<br>2002-2018	2002-2018
Finland	2002-2018<br>(50114 / 50085)	2006-2018<br>(6613 / 6577 / 6556)	2001-2018	2001-2018
France	2002-2018<br>(142438 / 143166)	2006-2018<br>(24306 / 24537 / 23398)	2001-2018	2001-2018
Germany	2002-2018<br>(103338 / 103509)	2006-2018<br>(16816 / 16846 / 16811)	2001-2018	-
Greece	2002-2018<br>(21894 / 22016)	2006-2018<br>(16650 / 16865 / 16810)	2001-2003<br>2004-2008*<br>2009<br>2010*<br>2011-2018	2001-2003<br><br>2009<br><br>2011-2018
Hungary	2002-2018<br>(22778 / 23097)	2006-2018<br>(6858 / 6957 / 6748)	2001-2018	2001-2018
Iceland	2002-2018<br>(2157 / 2318)	2006-2018<br>(294 /310 /105)	2001-2005*<br>2006-2009<br>2010-2013*<br>2014-2018	-
Ireland	2002-2018<br>(35329 / 35230)	2006-2018<br>(4482 / 4472 / 4217)	2001-2018	2004-2018
Italy	2002-2018<br>(56234 / 56503)	2006-2018<br>(18699 / 18802 / 18 833)	2001-2018	2005<br>2007-2008<br>2010-2018
Latvia	2006-2018<br>(1990 / 2006)	2006-2018<br>(1064 / 1072 / 1074)	2002<br>2004-2018	2002<br>2004-2018
Lithuania	2006-2018<br>(6523 / 6548)	2006-2018<br>(2091 / 2100 / 1933)	2006-2011*<br>2012-2018	2012-2018
Luxembourg	2002-2018<br>(5155 / 5197)	2006-2018<br>(747 / 750 / 644)	2001-2018	2001-2018
Malta	2002-2018<br>(3178 / 3176)	2006-2018<br>(909 / 909 / 908)	2007-2018	2001-2018
Netherlands	2002-2018<br>(67648 / 66388)	2006-2018<br>(9865 / 9806 / 9601)	2001-2018	2001-2002<br>2010-2018
Norway	2002-2018<br>(39646 / 40611)	2006-2018<br>(7180 / 7270 / 7076)	2001-2018	2001-2018
Poland	2002-2018<br>(15294 / 16405)	2006-2018<br>(5734 / 6189 / 6098)	2001-2002<br>2004-2005<br>2007-2018	2001-2002<br>2014-2018
Portugal	2002-2018<br>(46731 / 46714)	2006-2018<br>(16385 / 16384 / 15042)	2001-2006<br>2008-2018	2009-2018
Romania	2002-2018<br>(3232 / 3306)	2006-2018<br>(2067 / 2093 / 2039)	2009<br>2011-2018*	a)
Slovakia	2002-2005, 2011-2018<br>(7592 / 7519)	2006-2018<br>(3726 / 3715 / 3372)	2001-2009<br><br>2011*<br>2012-2018	2001-2002<br>2004-2009<br><br>2012-2018
Slovenia	2002-2018<br>(16733 / 16786)	2011-2018<br>(2920 / 2926 / 2872)	2001-2018	2001-2018
Spain	2002-2018<br>(81380 / 81951)	2006-2018<br>(15006 / 15082 /<br>14 828)	2001-2018 b)	b)
Sweden	2002-2018<br>(73200 / 77491)	2006-2018<br>(11186 / 11819 /<br>10 593)	2001-2018	2001-2018
United Kingdom	2002-2018<br>(139445 / 129069)	2006-2018<br>(22758 / 21224 /<br>21 827)	2001-2018	2013-2018

*: Total care data (countries reported community and hospital sector data as aggregated figures)
a): Romania reported antimicrobial consumption data for 2009 for the hospital sector, but these data were not included in the study

b): Spain reported reimbursement data on antimicrobial consumption during 2009-2015 then changed to reporting sales data from 2016 onwards. This resulted in a considerable technical increase in antimicrobial consumption between 2015 and 2016 due to the inclusion of non-reimbursed prescriptions. For this reason, Spain was not included in the sensitivity analysis (see Figure S1)
c): Spain reported antimicrobial consumption data for 2016-2018 for the hospital sector, but these data were not included in the study.

Methods

EU/EEA population-weighted mean percentages

Calculation of EU/EEA population-weighted mean is an established method to produce EU/EEA-level rates and are routinely used by ECDC as one of the main indicators for monitoring trends of both antimicrobial consumption and antimicrobial resistance [2,6].

For antimicrobial consumption, the population-weighted EU/EEA means were calculated by multiplying the antimicrobial consumption rate (DDD per 1 000 inhabitants per day) for each country by the corresponding national population, summing all the results and dividing this sum by the total population of participating EU/EEA countries. For antimicrobial resistance, the population weights represented the proportion of each individual country's population out of the total population of all included countries. The population-weighted EU/EEA mean percentages were calculated by multiplying the resistance percentage for each country by the respective population weight, and summing up the results. Weights were rescaled if data were not available for one or more countries. Annual population data were retrieved from the Eurostat online database [7].

The number of countries reporting data to ESAC-Net/ESAC and to EARS-Net/EARSS varied slightly over time (Table S1). No imputation of missing data was performed as national population weights were adjusted accordingly.

To assess the impact of missing data points in the ESAC-Net/ESAC and EARS-Net time series, data from the full database (as used in the main analyses) were plotted against data restricted to countries providing data for all years (see Figures S1 and S2).

Methods

Descriptive analysis of time trends

To determine significant time trends and points of change of each time series under study, a linear segmented regression analysis was performed [8] with the Joinpoint Regression Program [9].

Antimicrobial consumption rates and percentages of antimicrobial resistance were natural log transformed. Time was set as the independent variable. No covariates were used in the analyses. The minimum number of joinpoint was set as 0. The maximum number of joinpoint was set as one for *K. pneumoniae* data and two for *E. coli* and antimicrobial consumption, according to the length of the time series. The number of observations between joinpoints and to the last time series point were those proposed by the programme. We selected fitted auto-correlated errors models in order to account for autocorrelation of data from one year to the next. The modelling method used was Grid Search. Selection of the final model with the smallest number of joinpoints was based on the Bayesian Information Criterion (BIC) method, considering the model with the minimum value of BIC as the optimal model. For each detected time series segment, we calculated the Annual Percent Change and the corresponding 95% confidence interval. For describing the trends, we used the terms 'increase' or 'decrease' if the slope was statistically significant ($P \leq 0.05$) and we used the term 'stable' when slope was not statistically significant ($P > 0.05$).

Additional results

Sensitivity analysis to assess the impact of individual countries not reporting antimicrobial consumption and AMR data for all years during 2001-2018

To assess the impact of missing data points in the ESAC-Net/ESAC time series, data from the full database (as used in the main analyses) were plotted against data restricted to countries that continuously reported data for all years of the study period. As shown in Figure S1, there were few differences between time series that showed similar behaviours.

Likewise for EARS-Net/EARSS, data from all countries and laboratories (as used in the main analysis) were plotted against data restricted to countries that continuously reported data for all years of the study period and only including data from laboratories in these countries that provided data for >80% of the years. As shown in Figure S2, there were few differences between time series that showed similar behaviours.

FIGURE S1. Comparison of antimicrobial consumption data from all reporting countries (orange) with data restricted to countries that continuously reported data for all years of the study period (grey), by antimicrobial group and sector, EU/EEA countries, 2001 -2018

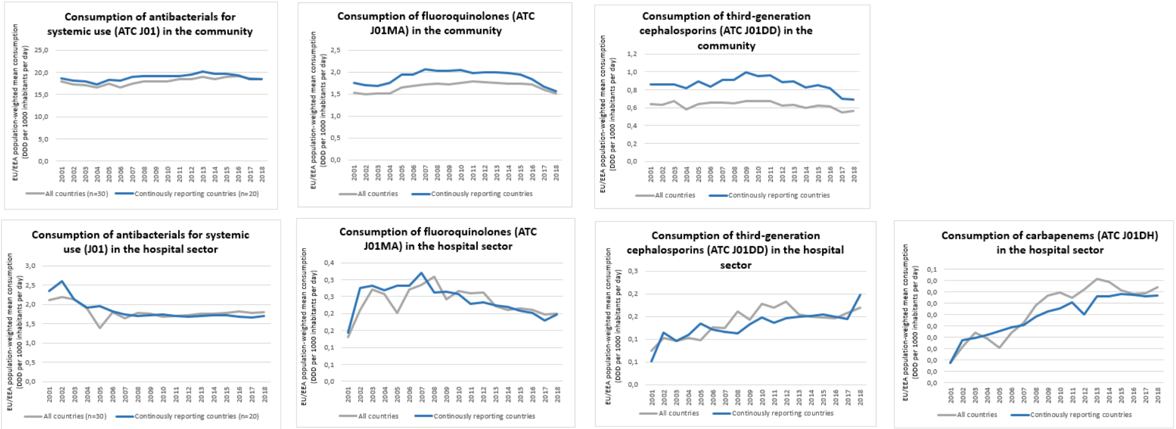

FIGURE S2. Comparison of antimicrobial resistance data from all countries and laboratories (orange) with data restricted to continuously reported data for all years of the study period and only including data from laboratories in these countries that provided data for >80% of the years (grey), by microorganism and antimicrobial group, EU/EEA countries, 2002-2018

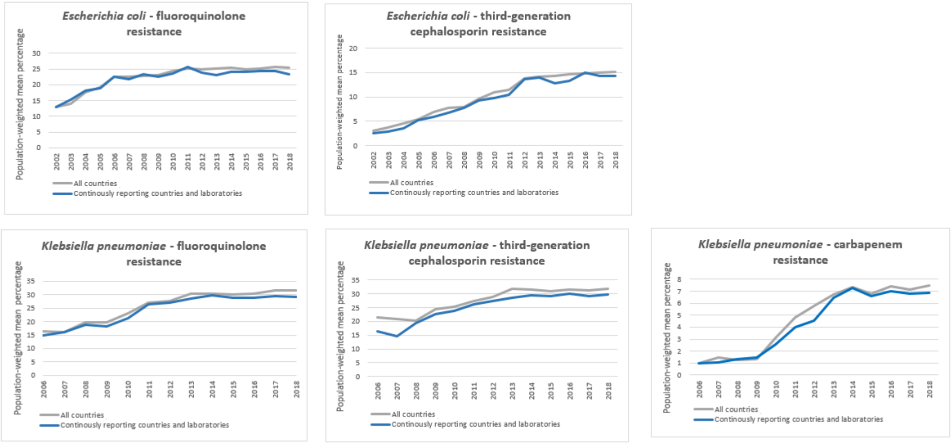

Total consumption of antibacterials for systemic use compared to consumption of fluoroquinolones, third-generation cephalosporins and carbapenems

The antimicrobial groups included in this study, i.e. fluoroquinolones (ATC group J01MA), third-generation cephalosporins (J01DD) and carbapenems (J01DH), were selected as they are of relevance to treat severe infections caused by *Escherichia coli* and *Klebsiella pneumoniae*. These antimicrobial groups corresponded to approximately 10.1%, 4.1% and 0.2% (mean proportion between 2001 and 2018) of the total consumption (reported from the community and the hospital sector combined) of antibacterials for systemic use (ATC group J01), respectively [2].

To put our results in perspective with total consumption of antibacterials for systemic use (ATC group J01), a separate analysis was performed and is presented here. Two segmented regression analyses were performed with the Joinpoint regression programme in the same manner as was done in the main analysis, for community consumption and for hospital sector consumption, respectively.

For community consumption, the overall trend for the whole period 2001 to 2018 was stable, but with three separate trend segments detected. After an initially stable trend between 2001 and 2004, an increasing trend was identified between 2004 and 2015, followed by a stable trend between 2015 and 2018 (Table S2).

For hospital consumption, the overall trend for the whole period 2001 to 2018 was decreasing, but with two separate trend segments detected. An initially decreasing trend between 2001 and 2005 was followed by a small, but statistically increasing trend between 2005 and 2018 (Table S2).

TABLE S2. Joinpoint regression analysis of trends: EU/EEA population-weighted mean consumption of antibacterials for systemic use (ATC group J01) expressed in defined daily doses (DDD) per 1000 inhabitants per day, per sector, ESAC-Net/ESAC, 2001–2018.

Segment	Lower endpoint		Upper endpoint		Annual percent change (APC)		
	Year	95% CI	Year	95% CI	APC	95% CI	P value
**Total consumption of antibacterials for systemic use (ATC group J01)**							
**Community sector**							
All years	2001	NA	2018	NA	+0.3	-0.3 to 0.8	0.351
Segment 1	2001	NA	2004	2002 to 2006	-1.6	-3.8 to 0.7	0.147
Segment 2	2004	2002 to 2006	2015	2010 to 2017	**+1.1**	**0.9 to 1,4**	**<0.001**
Segment 3	2015	2010 to 2017	2018	NA	-1.1	-3.4 to 1.2	0.303
**Hospital sector**							
All years	2001	NA	2018	NA	**-1.5**	**-2.1 to -0.8**	**<0.001**
Segment 1	2001	NA	2005	2004 to 2007	**-8.0**	**-10.6 to-5.4**	**<0.001**
Segment 2	2005	2004 to 2007	2018	NA	**+0.7**	0.3 to 1.1	**0.004**

CI: confidence interval; ESAC-Net/ESAC: European Surveillance of Antimicrobial Consumption Network/European Surveillance Antimicrobial Consumption project; NA: not applicable

Statistically significant trends are shown in bold.

REFERENCES

1. WHO Collaborating Centre for Drug Statistics Methodology. ATC Index with DDDs. Oslo, WHO 2019.

2. European Centre for Disease Prevention and Control. Antimicrobial consumption database (ESAC-Net). Available from https://www.ecdc.europa.eu/en/antimicrobial-consumption/surveillance-and-disease-data/database

3. European Centre for Disease Prevention and Control, European Food Safety Authority and European Medicines Agency. ECDC/EFSA/EMA second joint report on the integrated analysis of the consumption of antimicrobial agents and occurrence of antimicrobial resistance in bacteria from humans and food-producing animals – Joint Interagency Antimicrobial Consumption and Resistance Analysis (JIACRA) Report. Available from: http://ecdc.europa.eu/publications-data/ecdcefsaema-second-joint-report-integrated-analysisconsumption-antimicrobial

4. European Centre for Disease Prevention and Control. TESSy, The European Surveillance System – Antimicrobial resistance (AMR) reporting protocol 2019 – European Antimicrobial Resistance Surveillance Network (EARS-Net) surveillance data for 2017. Stockholm: ECDC; 2019.

5. European Committee on Antimicrobial Susceptibility Testing. EUCAST guidelines for detection of resistance mechanisms and specific resistances of clinical and/or epidemiological importance – Version 2.0. EUCAST; 2017. Available from: http://www.eucast.org/fileadmin/src/media/PDFs/EUCAST_files/Resistance_mechanisms/EUCAST_detection_of_resistance_mechanisms_170711.pdf

6. European Centre for Disease Prevention and Control. Surveillance of antimicrobial resistance in Europe 2017. Stockholm: ECDC; 2018.

7. Eurostat [internet]. Brussels: Eurostat; 2019. Accessed 10 October 2019. Available from: http://ec.europa.eu/eurostat.

8. Kim HJ, Fay MP, Feuer EJ, Midthune DN. Permutation tests for joinpoint regression with applications to cancer rates. Stat Med. 2000;19(3):335-51. Erratum: Stat Med. 2001;20(4):655.

9. Joinpoint Regression Program, Version 4.7.0.0 - April 2019; Statistical Methodology and Applications Branch, Surveillance Research Program, U.S. National Cancer Institute. Available from: https://surveillance.cancer.gov/joinpoint/
